# Osteosarcoma patient with Li-Fraumeni syndrome: the first case report in Vietnam

**DOI:** 10.3389/fonc.2024.1458232

**Published:** 2024-10-08

**Authors:** Thanh Thien Le, Tung Sy Ha, Linh Mai To, Quang Minh Dang, Hoa Thi Phuong Bui, Thanh Duc Tran, Phuong Thi Vu, Hoan Bao Giang, Dung Trung Tran, Xuan-Hung Nguyen

**Affiliations:** ^1^ Vinmec-VinUni Institute of Immunology, Vinmec Healthcare System, Hanoi, Vietnam; ^2^ Department of Medical Biology and Genetics, Hanoi Medical University, Hanoi, Vietnam; ^3^ Department of Biology, Hanoi University Science, Hanoi, Vietnam; ^4^ Department of Medical Genetics, Vinmec Hi-Tech Center, Vinmec Healthcare System, Hanoi, Vietnam; ^5^ Sarcoma Center, Vinmec Healthcare System, Hanoi, Vietnam; ^6^ Pathology Department, Vinmec Times City International Hospital, Vinmec Healthcare System, Hanoi, Vietnam

**Keywords:** Li-Fraumeni syndrome, Tp53 gene, whole-genome sequencing, genetic testing, ATMIN

## Abstract

Li–Fraumeni syndrome (LFS) is a hereditary disorder characterized by an increased risk of developing multiple early-onset cancers, primarily due to germline *TP53* mutations. Women and men with this mutation face lifetime cancer risks of 90% and 70%, respectively. This report describes the first documented case of LFS with clinical information in Vietnam involving a 9-year-old child diagnosed with osteosarcoma who had multiple first- and second-degree relatives with cancer. Whole-genome sequencing (WGS) revealed a heterozygous, pathogenic, autosomal dominant *TP53* variant NM_000546.6:c.733G>A (p.Gly245Ser) and a translocation in the 3’UTR of the *ATMIN* gene with unknown pathogenicity in both the patient and her mother. Sanger sequencing confirmed the presence of the *TP53* c.733G>A mutation, which was subsequently detected in extended family members. Of the 17 family members invited for testing, only 8, none of whom currently have cancer, agreed to participate: all tested negative for the mutation. This case highlights the importance of genetic testing for the early detection and management of cancers in LFS patients. It also underscores significant barriers to genetic screening in Vietnam, including limited access and the psychosocial consequences of testing, which emphasize the need for improved genetic counseling and surveillance strategies that are tailored to local contexts.

## Introduction

Li–Fraumeni syndrome (LFS) is a complex inherited genetic disorder characterized by a high predisposition to various types of cancer from an early age. The syndrome follows an autosomal dominant pattern of inheritance and predominantly manifests as five core cancer types: breast, adrenocortical, central nervous system (CNS), bone, and soft tissues (1). Individuals with LFS face a markedly earlier onset of cancer, with a 50% incidence for females under 31 years of age and males under 46 years of age and a higher lifetime cumulative risk, at 90% for females and 70% for males (2). Survivors of their first cancer have a 49% chance of developing another cancer after ten years (3), with the risk being even higher among survivors of childhood cancers (4).

The advent of next-generation sequencing (NGS) has further consolidated that *TP53* germline mutations is the leading molecular cause of LFS, occurring in approximately 75% of LFS patients (5). Among these, 77% are missense mutations that often result in a loss of function in *TP53* (6). The *TP53* gene encodes the protein p53, a transcriptional regulator known as the “guardian of the genome” (7). As a tumor suppressor, p53 maintains cell integrity by controlling essential functions such as DNA repair, growth arrest, autophagy, senescence, and apoptosis (8). When p53 surveillance activity is disrupted, genomic alterations accumulate, leading to cancer. Indeed, *TP53* mutations are found in 38% to 50% of almost all types of cancer, further highlighting the crucial role of this gene in tumor progression (6).

Despite the severe implications of LFS, the latest IARC Tp53 database (version R20) reports only approximately 1500 affected families, with potentially tens of thousands of cases remaining undetected (9, 10). NGS is vital for identifying *TP53* and expanding LFS screening. Gene panels targeting *TP53* exons are commercially available for patients suspected of having LFS. However, the adoption of this technology remains slow, especially in lower-middle-income countries such as Vietnam, where genetic screening for heritable cancers is not commonly practiced. Despite the notable prevalence of inherited cancer in Vietnam (11, 12), only one prior study has reported a germline *TP53* mutation, NM_000546.5:c.799C>T (p.Arg267Trp), without providing details on the associated clinical phenotype (13). This gap underscores the urgent need to investigate *TP53* mutation patterns in Vietnamese patients suspected of having LFS to characterize LFS in this underexplored population. Such research would provide valuable data to policymakers and the public, underscoring the importance of early screening for high-risk patients, which could lead to timely diagnoses and potentially improve survival rates.

In this report, we described the case of a 9-year-old Vietnamese girl who presented with left distal femur pain and was later diagnosed with osteosarcoma. The patient had an extensive familial history of cancer, including 2^nd-^ and 3^rd^-degree relatives with multiple malignancies. Whole-genome sequencing (WGS) conducted on the patient and her immediate family identified a *TP53* missense mutation, and a novel translocation mutation inherited from her mother. Further genetic screening using Sanger sequencing was offered to the patient’s extended family to assess their risk. This study represents the first reported case of LFS in Vietnam, underscoring the vital role of genetic testing in managing heritable cancers such as LFS.

## Methods

### Study population

The patient (proband) was referred to Vinmec International Hospital (Hanoi, Vietnam) for surgery as part of her osteosarcoma treatment. Surgical excision of her tumor was performed according to a routine protocol. During the evaluation, a significant family history of cancer was uncovered. Post-recovery, upon gaining mobility, we approached her legal guardian (mother) for consent to include her in our genetic study. Following the discovery of a heritable mutation, extended genetic testing was offered to her broader family.

### Whole-genome sequencing

WGS was conducted at no cost by Inocras (San Diego, USA) as part of their Clinical Excellence Program. DNA was extracted from blood samples using a Qiagen DNA Blood Kit (Qiagen, Maryland, USA). The DNA concentration was measured with a Qubit instrument (Thermo Fisher, Massachusetts, USA). The samples were then sent to Inocras for sequencing. The RareVision™ system (Inocras, San Diego, USA) was used for library preparation, genome sequencing, analysis, and interpretation. In brief, DNA library preparation was performed using Watchmaker DNA Library Kits (Watchmaker Genomics, Boulder, USA), and the DNA was subsequently sequenced on an Illumina NovaSeq 6000 platform (Illumina, San Diego, USA). The raw sequences were subjected to quality control and aligned to the GRCh38 human reference genome using BWA-MEM (14). Single-base substitutions and short indels were called using HaplotypeCaller2 (15) and Strelka2 (16). For complex structural variations, mutation calling was performed using Manta (17).

### PCR and Sanger sequencing

Based on the WGS results, we designed PCR primers targeting regions 350 bases upstream and downstream of the point mutation using Primer Blast (18). Primers were checked for GC content, annealing temperature, and self-complementarity using Primer Stats (19). The PCR primers used were PCR Forward – CCATCCTGGCTAACGGTGAA and PCR Reverse – AGAGGTCCCAAAGCCAGAGA. To ensure accurate Sanger results, we designed another forward primer used in Sanger sequencing to avoid poly-A regions upstream of the point mutation: Sanger Forward – CTCCCCTGCTTGCCACAGGT. After purification, the PCR products were sent to PhuSa Genomics for Sanger sequencing using Sanger forward and reverse PCR primers. The Sanger results were aligned using Unipro UGENE (20).

## Case presentation

### Clinical information

**Figure 1:**
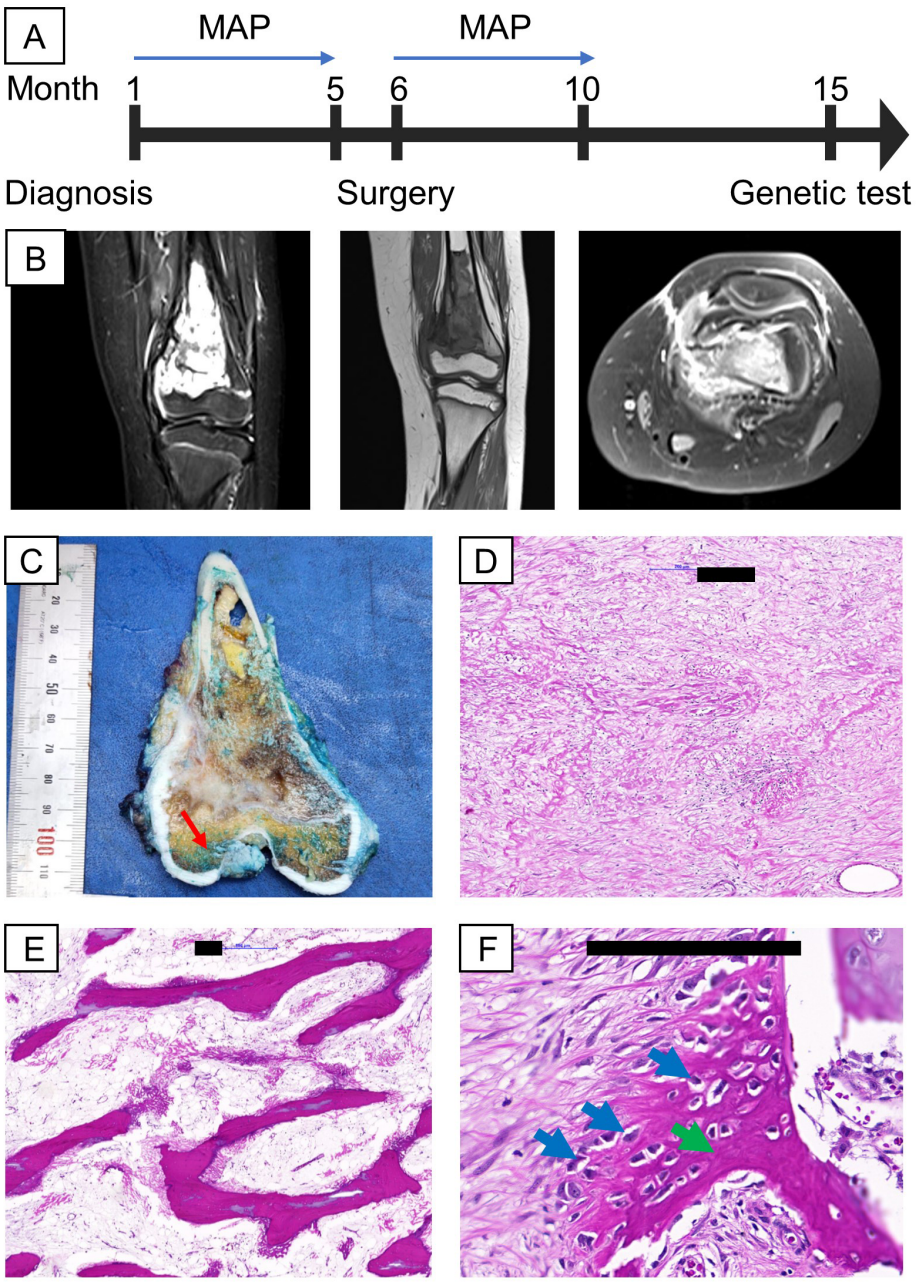
Clinical information, including MRI and H&E images of the proband’s tumor. **(A)** Timeline of relevant episodes of care; **(B)** MRI showed the presence of the tumor in the left distal femur (from left to right: 2 cross sections coronal view of the femur, axial view of the femur; **(C)** On gross examination, a tan-white, irregular tumor was found intramedullary in the metaphysis region, suspicious for cortical destruction (red arrow); **(D, E)** Representative sections of the gross tumor revealed good response to chemotherapy on microscopic examination characterized by cell dropout, densely sclerotic bony trabeculae and sclerosing reaction; **(F)** A small portion of the tumor are non-responsive, shown by viable tumor cells (blue arrows) surrounded by eosinophilic osteoid (green arrow). Black scale bars denote 200 µm.

A 9-year-old female was clinically diagnosed with osteosarcoma in September 2022 at a local hospital following a bone biopsy that identified conventional osteosarcoma. She underwent two cycles of combination chemotherapy, methotrexate, doxorubicin, and cisplatin (MAP), at Vietnam National Cancer Hospital before being referred to Vinmec International Hospital for further treatment in January 2023 (**Figure 1A**). Clinical examination revealed a firm, swollen mass in her left distal femur. The tumor, measuring 7x4.5x3.5 cm on MRI, was located in the distal femur within the medullary canal, broke through the bone cortex, and invaded the surrounding soft tissues without affecting the nearby neurovascular bundle (**Figure 1B**). No distant metastasis was detected via lung SPECT/CT. The final diagnosis was stage IIB osteosarcoma of the left distal femur. Her surgery was conducted in February 2023 and involved wide resection of the tumor and reconstruction using an expandable prosthesis. Pathological examination of the resected tissue indicated 98% necrosis, with the margins free of tumor cells and minimal viable tumor cells remaining (**Figures 1C–F**). Postsurgery, the patient resumed chemotherapy with the MAP regimen, completing a total of 29 weeks of treatment by June 2023. She responded favorably to the treatment, regaining the ability to walk.

### Genetic counseling and testing

**Figure 2:**
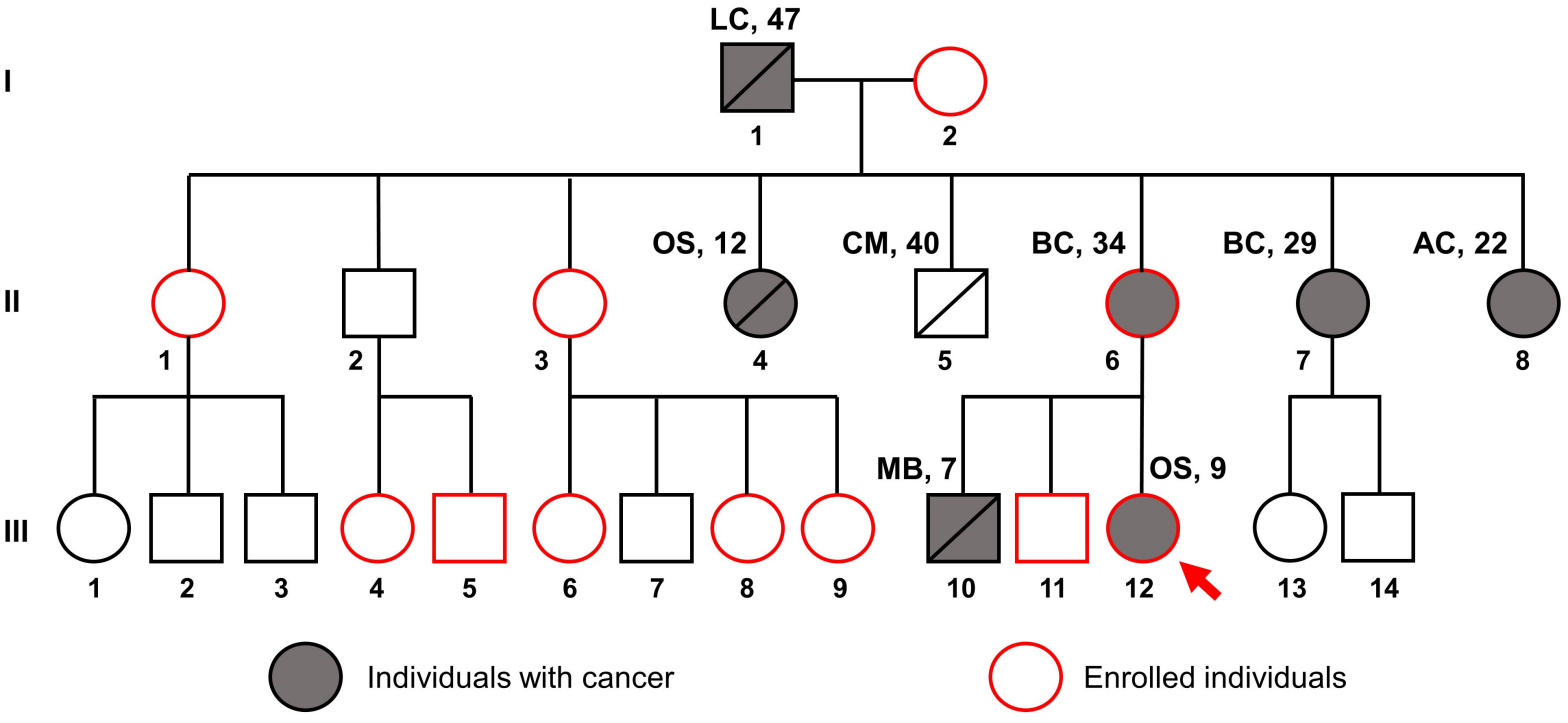
Pedigree of the family suspected of Li-Fraumeni syndrome. The red arrow indicates the proband, the gray filling indicates individuals with cancer, and the red outline indicates individuals enrolled in this study. Disease names are followed by age at diagnosis. LC, Lung cancer; OS, Osteosarcoma; CM, Cerebrovascular Malformation; BC, Breast Cancer; AC, Astrocytoma; MB, Medulloblastoma.

The patient presented with a significant family history of cancer, including a brother who died from medulloblastoma at the age of 7, a mother with breast cancer at 34, and various other malignancies among relatives (**Figure 2**), fitting the classical criteria of LFS. While osteosarcoma is prevalent in LFS, accounting for 3-16% of cases, the proband developed cancer at a much earlier age than the median age of 14 among LFS individuals (21). Consequently, she was referred for genetic testing in August 2023 to evaluate for hereditary cancer syndromes. WGS was performed on the proband (III.12), her brother without any present signs of cancer (III.11), and her mother with breast cancer (II.6).

**Figure 3:**
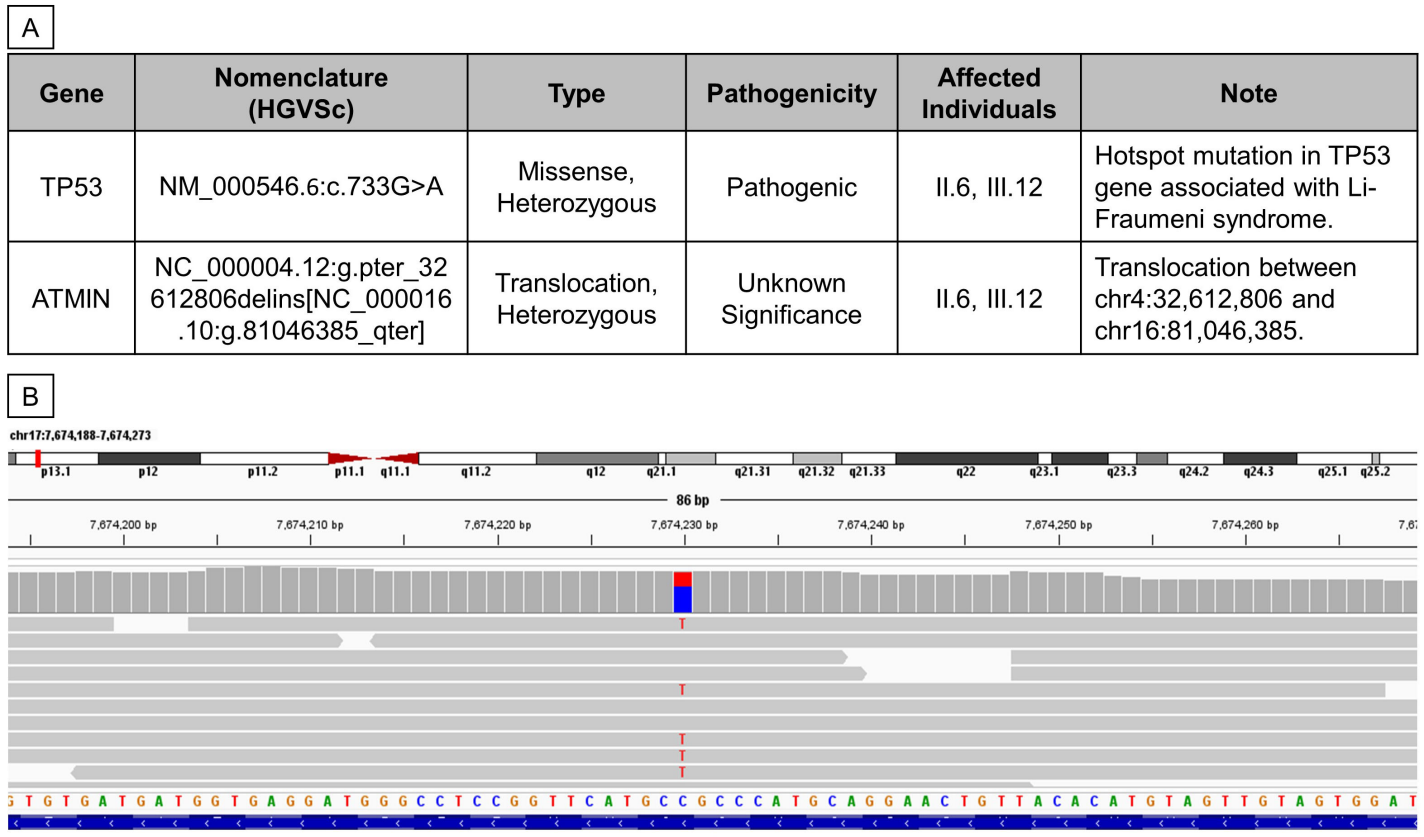
Whole Genome Sequencing revealed two germline mutations in II.6 and III.12. **(A)** Table summarizing germline variants found from WGS analysis. **(B)** Reads alignment view using Integrative Genomics Viewer (Thorvaldsdóttir et al., 2013) of patient III.12.

The patients’ genomes were sequenced with an average coverage of 20x. WGS analysis detected a heterozygous, pathogenic, autosomal dominant variant, NM_000546.6:c.733G>A (p.Gly245Ser), in the *TP53* gene (**Figure 3A**), present in both the proband and her mother but absent in her brother. The read alignment showed a heterozygous C>T mutation on the forward strand, translating to a G>A mutation on the reverse strand, where the *TP53* gene is located (**Figure 3B**). Located in exon 7, this G>A mutation substitutes the amino acid glycine (GGC) for serine (AGC) - p.(Gly245Ser), altering the protein structure. This change results in a loss of wild-type *TP53* transcriptional activity and a gain of function, enhancing Akt signaling and transformation in cell cultures (22–24). This dominant-negative missense TP53 variant, known for its high penetrance and frequent occurrence in families with childhood cancers, was first reported by Srivastava et al. in 1990 as being linked to LFS (25). It is classified as pathogenic or likely pathogenic in the ClinVar database (26–29).

Additionally, the proband and her mother were found to carry another heterozygous variant, NC_000004.12:g.pter_32612806delins[NC_000016.10:g.81046385_qter], which involves a 3’-to-5’ translocation between chr4:32,612,806 and chr16:81,046,385 (**Figure 3A**). The breakpoint at chr16:81,046,385 lies within the 3’UTR of the ataxia telangiectasia mutated interactor (*ATMIN*) gene. This particular mutation has not been previously reported, and its pathogenicity remains uncertain; hence, it was not further validated in this study.

**Figure 4:**
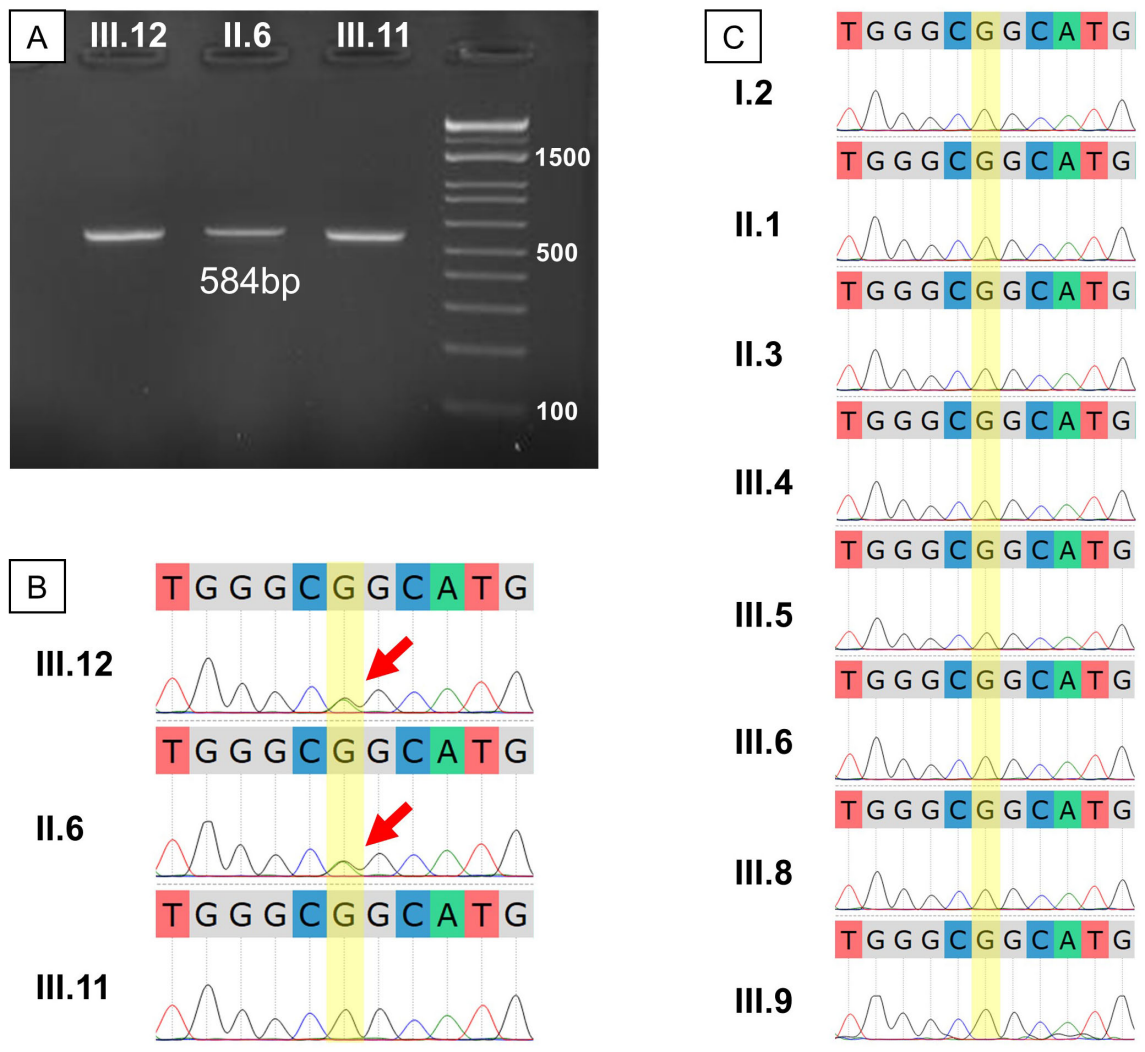
Genetic result validation and extended testing for the studied family. **(A)** Gel electrophoresis of PCR products showed the expected fragment size of about 584 bp; **(B)** Sanger sequencing of the proband’s immediate family showed a heterozygous G>A mutation in III.12 and II.6; **(C)** Sanger sequencing for the extended family showed no other G>A mutation carrier among those who consented to the test.

Sanger sequencing was employed to confirm the WGS findings. Amplification of the relevant *TP53* gene region yielded the correct band size (**Figure 4A**). Sequencing revealed a double G-A peak at the mutation site, indicative of a heterozygous mutation in both the proband and her mother but not in her brother (**Figure 4B**). We then invited the entire extended family to join the study for free screening with Sanger sequencing. Of the 17 members invited, 8 healthy individuals with no first-degree relative with cancer agreed to enroll (**Figure 2**). Analysis of the *TP53* c.733G>A mutation in these extended family members revealed that none were carriers of the mutation, as indicated by a single G peak at the expected location (**Figure 4C**).

## Discussions

### Clinical and genetic manifestations

Our case study marks the first reported instance of LFS in Vietnam with comprehensive clinical and familial documentation. Although the recognition of LFS and other inherited cancer syndromes is increasing in Vietnam, genetic testing remains underutilized (12). We identified a pathogenic *TP53* mutation, c.733G>A, a well-documented hotspot within the DNA binding domain of the p53 protein. This mutation is associated with various types of cancer, including breast cancer, osteosarcoma, adrenocortical carcinoma, and CNS cancers, according to the ClinVar database. Breast cancer, representing 27% to 31% of LFS-associated cancers, has a median age at diagnosis of 33 years (21); in our study, two patients were diagnosed at ages 29 and 34. Osteosarcoma, which accounts for 3%-16% of LFS cancers, typically presents at a median age of 16 (10); however, in our studied family, members were diagnosed at ages 9 and 12. For CNS tumors, which are reported in 9%-14% of LFS patients with a median onset age of 16 years, our findings included a patient who was diagnosed with astrocytoma at 22 years of age and another patient who was diagnosed with medulloblastoma at 7 years of age (**Figure 2**).

We also detected a translocation in the 3’UTR of the *ATMIN* gene. *ATMIN* was initially identified as a DNA damage response protein involved in DNA repair processes, particularly through non-homologous end joining and base excision repair, functioning in conjunction with the ataxia telangiectasia mutated (*ATM*) protein (30–32). Under stress conditions, *ATMIN* and *ATM* kinase act as cell cycle checkpoint regulators, helping to mitigate the accumulation of DNA damage (31, 33, 34). Cells lacking *ATMIN* exhibit reduced ATM activation, and conversely, cells deficient in ATM show diminished *ATMIN* expression (33, 35). Despite this reciprocal interaction, while ATM germline mutations are well-established as a genetic modifier in LFS and other cancer predisposition syndromes (36, 37), much less is known about the role of its cofactor *ATMIN* in human cancer.

According to the ClinVar database, there are 130 reported cases of *ATMIN* germline mutations, with most classified as variants of uncertain significance. In lung adenocarcinoma patients, *ATMIN* is frequently lost, and its low expression is associated with poorer prognosis (38). In preclinical models, mice lacking *ATMIN* develop B-cell lymphomas (32), or exhibit a higher tumor burden and grade in lung cancer (38). Patients with mutated *TP53* and *ATMIN* signaling, both of which are vital for maintaining genetic integrity, could potentially accelerate the accumulation of DNA damage and contribute to the observed earlier onset of cancer. However, the exact implications of *ATMIN* mutations in cancer predisposition still require further investigation to be fully understood.

Our findings underscore the significant advantages of WGS in capturing genetic variants, offering a notable benefit over targeted panel testing, which may miss novel mutations linked to heritable cancers. Indeed, the heterogeneity of LFS (9), which suggests it is an oligogenic disease influenced by multiple genes, further supports the use of WGS in these cases. However, WGS is not without its limitations. While it excels at providing a broad genetic overview, WGS is more costly than panel testing and may not detect complex rearrangements or mosaic variants due to the inherent limitations of short-read sequencing technologies. Additionally, the complexity of analyzing multiple variants, especially in patients with compromised DNA repair mechanisms like those with LFS, adds another layer of difficulty. Instead, a targeted gene panel approach might be more practical for large-scale screening, especially in resource-limited settings like Vietnam. Consequently, careful consideration is needed when selecting screening methods, balancing their capabilities against their limitations.

### Possible early-onset cancer and screening

A possible trend in the studied family is that cancer diagnoses appear at progressively earlier ages in successive generations. Specifically, the age at diagnosis was 47 for the first generation; 34, 29, 22, 12 for the second generation; and 9, 7 for the third generation. This trend, sometimes observed in LFS family (39–41), is concerning since survivors of childhood cancers have a substantially greater risk of developing a second cancer (3.2 incidences per 100 person-years) than their older counterparts (2.0-2.7 incidences per 100 person-years) (4). Such data highlight the critical need for proactive and early screening measures for *TP53* carriers to lessen the impact of cancer on families.

Surveillance guidelines, such as those recommended by the “Toronto Protocol”, suggest rigorous evaluations (42, 43). This includes comprehensive physical examination and ultrasound every 3 to 4 months for children under 18 years old; physical examination every six months, ultrasound and dermatologic examination annually for individuals 18 years or older; and upper endoscopy and colonoscopy every 2 to 5 years for individuals 25 years or older. Women are suggested to undergo clinical breast examination every 6 to 12 months from 20 to 25 years of age, annual breast MRI from 20 to 30 years of age, and annual mammogram and breast MRI from 30 to 75 years of age. After cancer has been diagnosed, an annual neurologic exam and whole-body MRI, including brain MRI, are recommended.

The effectiveness of the Toronto Protocol in detecting cancer early and prolonging survival for LFS patients has been well documented. Villani et al. conducted an observational study of 18 *TP53* mutation carriers and found that those adhering to the protocol had their tumors identified at a lower grade or premalignant stage, compared to higher-grade and stage tumors in patients not following the protocol (44). A subsequent 11-year follow-up study further confirmed that compliance with this screening strategy leads to better outcomes, with a 5-year survival rate of 88.8% for those under surveillance, compared to 59.6% for individuals who were not monitored (43).

### Barriers to screening adherence

In our study, despite receiving genetic counseling and understanding the benefits of genetic screening, some patients opted not to participate due to health information avoidance. This behavior, where individuals deliberately avoid information that could cause them distress, is observed in conditions like LFS (45, 46). The anxiety associated with the prospect of lifelong, costly surveillance after a positive result leads some patients to prefer remaining uninformed about their genetic risks. This is particularly true in Vietnam, where such screenings are often considered elective and are not covered by insurance, making cost a significant barrier to adherence (47). However, cost is not the only obstacle, as some patients in our study still refused screening even when it was offered for free. The social stigma associated with inherited diseases further exacerbates this avoidance; only healthy family members agreed to screening, while those with a cancer diagnosis or close relatives affected by cancer declined participation. Similar behaviors have been observed in other screening studies conducted within Vietnamese and comparable Asian cohorts, where the primary reasons for rejecting screening are psychological (12, 48, 49). This underscores the necessity for enhanced education and support systems that address the psychological and economic challenges associated with genetic testing in Vietnam, as well as broader public health initiatives aimed at reducing the stigma linked to genetic conditions.

Countries with advanced genetic care systems, such as those in North America and Europe, provide successful models for overcoming these barriers. Initially, these regions faced concerns about genetic discrimination and privacy (50), but over time, the public began to recognize the significant benefits of genetic testing for early cancer detection (51). These countries have integrated genetic and imaging screenings into routine healthcare, often covered by insurance, thereby reducing the economic burden on patients. Additionally, education and social science have been integral to their screening strategies, making patients more aware of the benefits of early detection, which can lead to more effective treatment and improved survival rates. Public education, genetic counseling, and protective policies have played crucial roles in changing perceptions and increasing participation (2, 46). These insights are essential for regions like Vietnam to advance their genetic screening programs and improve patient outcomes. Thus, concerted efforts are necessary to raise disease awareness and develop more cost-effective, less burdensome cancer screening technologies or strategies to improve public perception and support for hereditary conditions.

In conclusion, the genetic screening conducted in this study has been invaluable for the patient’s family by identifying at-risk members, providing a clear path for disease management, and alleviating the anxiety of non-carriers. Our case report not only adds to the global body of knowledge on Li-Fraumeni syndrome but also emphasizes the existing disparities in genetic screening and the critical need for tailored cancer prevention and management strategies in Vietnam. Understanding the patterns of TP53 mutations in this underexplored population could pave the way for more targeted and cost-effective testing approaches, thereby improving the accessibility of such screenings. Furthermore, by integrating genetic counseling and addressing the psychosocial dynamics within affected families, we can enhance the effectiveness of surveillance programs and improve overall outcomes for patients with hereditary cancer syndromes.

## Data Availability

The original contributions presented in the study are publicly available. This data can be found here: https://www.ncbi.nlm.nih.gov/bioproject/PRJNA1163004/, ascension number PRJNA1163004.
